# Mechanical Properties of Protective Coatings against Marine Fouling: A Review

**DOI:** 10.3390/polym13020173

**Published:** 2021-01-06

**Authors:** Alessandro Pistone, Cristina Scolaro, Annamaria Visco

**Affiliations:** 1 Department of Engineering, University of Messina, Contrada Di Dio, I-98166 Messina, Italy; cristina.scolaro@unime.it; 2 Institute of Polymers, Composites and Biomaterials—CNR IPCB, Via Paolo Gaifami 18, 9-95126 Catania, Italy

**Keywords:** antifouling, anticorrosive, epoxy coating, siloxane coating, hybrid coating, mechanical properties, adhesion, wet ability

## Abstract

The accumulation of marine organisms on ship hulls, such as microorganisms, barnacles, and seaweeds, represents a global problem for maritime industries, with both economic and environmental costs. The use of biocide-containing paints poses a serious threat to marine ecosystems, affecting both target and non-target organisms driving science and technology towards non-biocidal solutions based on physico-chemical and materials properties of coatings. The review reports recent development of hydrophobic protective coatings in terms of mechanical properties, correlated with the wet ability features. The attention is focused mainly on coatings based on siloxane and epoxy resin due to the wide application fields of such systems in the marine industry. Polyurethane and other systems have been considered as well. These coatings for anti-fouling applications needs to be both long-term mechanically stable, perfectly adherent with the metallic/composite substrate, and capable to detach/destroy the fouling organism. Prospects should focus on developing even “greener” antifouling coatings solutions. These coatings should also be readily addressable to industrial scale-up for large-scale product distribution, possibly at a reasonable cost.

## 1. Introduction

Submerged boat marine structures are subject to the formation and accumulation of biofouling in its various forms over time. Biofouling increases progressively, becoming diversified and serious [1]. It a problem because it hinders the normal hydrodynamic movement of a boat. Consequently, a higher fuel consumption is necessary to obtain the required propulsive power [2] with adverse effects in the marine environment due to increased amount of fuel oils and residues into the sea than normal [3].

**Scheme 1:**
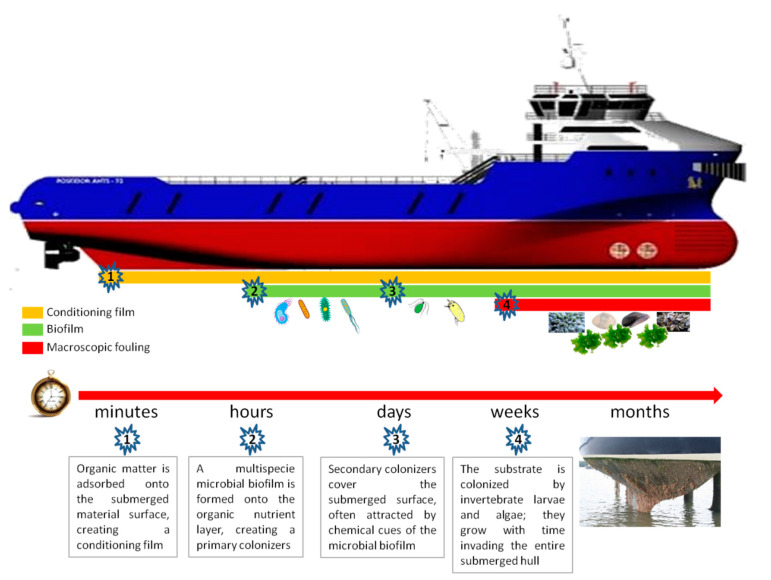
Mechanism of fouling formation onto the surface of submerged structures.

A clean surface immersed in natural seawater immediately starts to adsorb a molecular ‘conditioning’ film primarily consisting of dissolved organic material, which represents the nutrient for the subsequent colonization by bacterial biofilms (microfouling) [4,5,6,7]. Bacterial biofilm formation is then followed by spores of macroalgae (seaweeds), fungi, and protozoa (soft macrofouling), followed in turn by larvae of invertebrates, such as barnacles (macrofouling) (Scheme 1).

Antifouling paints represent the solution to inhibit the development and growth of biofouling on substrates in contact with seawater and constitute a real protective coating.

An increasing interest in fundamental science behind the processes involved in biofouling and in the development of novel coatings technologies re-started in the last years. The main driver was legislation that has outlawed the highly effective antifouling paints based on metal biocides (mainly tributyltin, copper-, and zinc-based systems) posing a stricter evaluation and regulatory regime on the use of alternative biocides. Long-term use of such metal biocides revealed in fact a host of unintended environmental impacts due to buildup phenomena in harbors and marinas; tributyl tin was banned in 2008 by a convention set by the International Maritime Organization [8].

Industrial and academic research is trying to find more ecological (“green”) alternatives to traditional technologies, which are based on metal biocides. This goal is being sought not only through alternative non-metallic biocides [9], but also through the development of non-toxic, i.e., biocide-free, coatings. This last type of coatings is based on the study of the physical-chemical properties of the surface of the materials to achieve two objectives. The first concerns the prevention of the possibility of attack by marine organisms (antifouling approach) by, for example, the formation of highly hydrated layer surrounding amphiphilic moieties able to modulate antifouling properties, where hydration repulsion arising from the water clustering in hydration shells surrounding charged groups may suppress the adsorption onto surfaces of unwanted molecules [10,11]; while the second concerns the reduction of the adhesion force after the attack (fouling release approach) so that they are easily removed by the shear forces generated by the movement of the ship or by gentle mechanical cleaning devices from surfaces coated with silicone based systems modified by copolymerization or mixing with organic or inorganic additives [12,13,14].

In both cases, the goal is achieved through an interdisciplinary work between skills in the biological, materials science, and engineering fields. The control and modification of the physico-chemical properties of the coating materials (for example, elastic modulus, coefficient of friction) is of fundamental importance to ensure that the surface becomes inhospitable for the marine organism. In this way, the forces of intermolecular interaction between the surface and the encrusting organism are weakened, favoring the detachment of the organism itself.

The design of a truly universal coating must be also take into account that the coating must work in a complex and varied marine environment. Marine vessels often traveling across continents are exposed to different fouling organisms with different mechanisms of attachment in different environmental conditions. Vessel operations are also a key factor in designing a successful coating. The operational profiles can range from constant dynamic conditions (e.g., under high speeds navigation) to static conditions (e.g., during extended periods of docking) [15]. Furthermore, the coating should be durable for years (or decades) without the need for constant reapplication.

**Figure 1:**
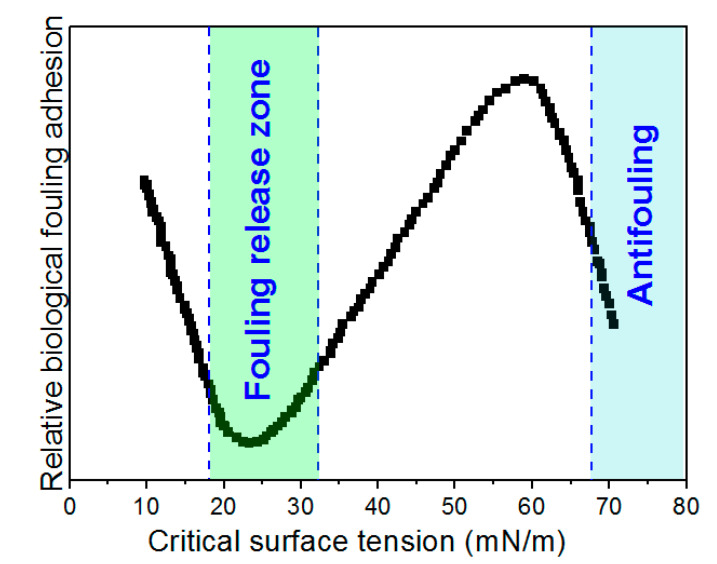
A Baier curve plotting the degree of fouling versus critical surface tension (Adapted with permission from [17]).

One of the most important criterion in the design of antifouling or fouling release coatings is surface chemistry. Hydrophilic coatings, with high surface energies like water (72 mN/m) and high affinity for polar molecules of water, removes any thermodynamic advantage from the adsorption of biomolecules, because it is energetically favorable for the surface to remain in contact with water rather than an amphiphilic biomolecule like a protein. On the other side, hydrophobic surfaces with low–surface energies materials are designed to minimize interactions with biomolecules by eliminating the ability for strong polar interactions (like hydrogen or ionic bonding). Thus, biomolecules and bio-organism can adhere only very weakly to these surfaces, making their removal easier by a fouling release mechanism. The relationship between surface energy and fouling release ability is well established and described by the Baier curve (Figure 1). Baier found that there was a minimum between 20 and 30 mN/m that gave optimal fouling release behavior while antifouling works at values more than 70 mN/m [16,17].

Also, the physical properties of a surface are significant. Barnacle removal can be modeled by the adhesion of a rigid solid attached to an elastomeric film, where the critical pull-off force is proportional to (Eγ)^1/2^, where γ is the surface energy of the material and E is the elastic modulus. As the modulus of the material is lowered, the critical pull-off force is minimized [18]. Film thickness (t) has also been identified as an important factor in fouling release materials with adhesion strength decreasing with the increase of film thickness as a function of (t)^−1/2^ [14]. 

Another important factor is surface roughness that is known to increase settlement and adhesion strength of biomolecules, including the proteinaceous adhesives used by many marine organisms [19]. All the above-mentioned properties, match well with polydimethylsiloxane (PDMS) and fluorinated materials, which are explored extensively as fouling release coatings [20]. For these reasons, the design of efficient fouling resistant coating is carried out taking in mind surface chemistry, mechanical properties, durability, and substrate attachment. 

About the mechanical properties, the most common tests for the coating’s adhesion measurements are: The cross-hatch tape adhesion test (following the ASTM-D3359-09e2 or ISO 2409:2013 standard), the peel test at 180° (ASTM D903 or ISO11339:1990), peel test at 90° or T-peel test (ASTM D6862-11:2016 or ISO1133-9:2010), the pull off test (ASTM D4541-02 or ISO4624:2016), pseudo-barnacle test (ASTM D5618), both in normal conditions and under simulated weathering conditions. An example of test under simulated weathering conditions is the salt spray test (ASTM B 117-03 or ISO9227) [21,22,23].

In this review, the developments in the more recent years on the mechanical properties of hydrophobic coatings, have been collected and presented. The review is organized in different chapters, depending on the coating chemistry. Coatings are mainly based on epoxy and siloxane resin, or epoxy modified poly-siloxane based resins. Polyurethane coatings have been considered as well. This review can help to evidence both the positive as well as the negative aspect of each resin, and therefore the necessity of the scientific community to find new solutions of coatings, such as the hybrid coatings here discussed as “other systems”, to overcome the limits of each resin. We considered the mechanical properties of coatings, in terms of coating adhesion toward the metallic/composite substrate. Contact angle values have been considered as well to evaluate the hydrophobic/hydrophilic behavior of each coating.

## 2. Silane-Based Coatings

In the last years, the number of scientific studies related to silane-based materials for antifouling coatings has increased exponentially. Silane-based coatings offer characteristics and performance not found in traditional organic coatings. They can be easily combined with organic monomers and polymers or inorganic fillers to develop specialized blends with the functionality of both organic and non-organic materials [24].

While possessing good mechanical properties in terms of adhesion, elasticity, and impact resistance, further studies were needed to specifically improve other characteristics, such as hydrophobic or hydrophilic properties. Therefore, the polydimethylsiloxane matrix was modified with inorganic or metallic fillers (such as titania, silica, ceria, zinc oxide, or silver) to change its hydrophilic/hydrophobic character and increase its resistance to fouling. 

Table 1 resume all wet ability and mechanical adhesion test results of the silane-based coatings analyzed in this paragraph. 

The polydimethylsiloxane loaded with fluoroalkyl silane (FAS) functionalized with cerium dioxide (CeO_2_) was applied by spray on the supports; the contact angle with water was 161 ± 2°, indicating excellent hydrophobic behavior. The super hydrophobicity is due to its surface consisting of a multi-level structure in micro and nano scale [25]. The same authors (An et al.) also point out that the air pockets formed in the grooves by the micro-nano structure help to reduce the contact area of the water drop on the surface, thus improving the anti-wetting properties. In the case of the FAS-CeO_2_/PDMS coating, the superhydrophobic properties are the result of both the low interfacial energy of the FAS-CeO_2_ particles and the roughness of the micro-nano scale. 

As known, crosscut and adhesion test evaluation ranges between 0B (which represents the complete detachment of crosscut patterns) and 5B, which represents no detachment of crosscut patterns. Cross-cut adhesion test showed that the coatings possessed also outstanding adhesion strength with about 15% of the sample surface area damaged obtaining a classification of level 2 (which corresponds to 4B, for the ASTM3359-09e2 and to ISO1, for the ISO2409:2013 standard). Considering that the levels are from 5B (or ISO 0), which is the highest level, to the 0B or ISO5, which is the lowest one, the level 4b represents a quite high adhesion level. The compatibility and synergistic effects between FAS-CeO_2_ nanoparticles and PDMS was reported as the main reason for the enhanced adhesiveness due to the significant reduction of cracking of the coating due to stress. The mechanical stability of superhydrophobic coating was evaluated by the sandpaper abrasion test and changes in water contact/sliding angles for different sandpaper abrasion cycles. The water contact/sliding angle of coating surface changed within a certain range (CA > 150°, SA < 6°) during the abrasion test and the FAS-CeO_2_/PDMS coating maintained a good wear resistance even after 30 cycles. A new micro-scale rough structure was formed after the destroying of the original micro-nano scale structure of the surface due to sandpaper abrasion. Since the PDMS showed good adhesive interaction with FAS-CeO_2_ nanoparticles, the coating can withstand up to 30 abrasion cycles. Therefore, the coating demonstrates good mechanical stability in addition to the excellent super hydrophobicity discussed above.

**Figure 2:**
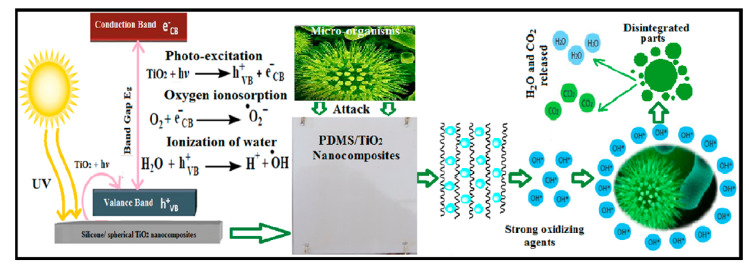
Scheme of fouling resistance mechanism under UV irradiation in PDMS-TiO_2_ coating (permission of ref. [26], https://s100.copyright.com/AppDispatchServlet?publisherName=ELS&contentID=S2352340916305091&orderBeanReset=true).

TiO_2_ spherical single crystal photo-nanofillers were embedded inside PDMS matrix; the composites showed increased fouling resistance (FR) after stimulation through UV radiation exposure (schematized in Figure 2), [26]. Impact, crosscut, and T-bending tests were performed on PDMS/TiO_2_ nanocomposites applied on a steel panel previously treated with an epoxy resin primer coat and an epoxy/silicone (50:50) tie coat. Authors found that oxidation and reduction occur at the surface of TiO_2_ nanoparticles with beneficial effect on the wetting characteristics and super hydrophilic performance. Impact test showed no cracks formation, indicating the high elasticity, and flexibility of the tested nanocomposite coatings. The cross cut test was carried out without resulting visible adhesion defects and no intrusion was identified under a magnifying glass in any of the tested paints after penetration and bending on a < 5 mm cylindrical spindle. 

Wettability test after UV irradiation test showed contact angle close to 10° for the sample loaded with 0.5 wt% of TiO_2_ indicating super-hydrophilicity and increased self-cleaning ability without resulting visible adhesion defects after cross-cut test.

Wu et al. [27] investigated a new method of combining both low and high surface energy SiO_2_ nanoparticles in order to prepare superhydrophobic and super-oleophobic coating based on 1H,1H,2H,2H-Perfluorooctyltriethoxysilane (PFOTES) on glass fiber reinforced epoxy (GFRE) substrates. The synthesized PFOTES-SiO_2_ nanoparticles were used as low surface energy fillers while pristine SiO_2_ nanoparticles as high surface energy ones. GFRE substrates were treated by spray coating of solution of PFOTES-SiO_2_ and SiO_2_ with different molar ratio. 

The authors demonstrated that the different oleophobicity can be adjusted by optimizing the molar ratio of the low and high surface energy nanoparticles. This has a direct effect on the surface roughness and surface energy of the coating surface. In particular, the coating obtained with a 2:4 molar ratio of PFOTES-SiO_2_ is not brittle and has good adhesion to the substrate. With this optimal coating formulation, pencil scratch tests were obtained with the resulting value of 8 H, cross-cut adhesion of 5B, and nanoindentation hardness of 0.24 GPa. It should be remembered that the pencil test measures the scratch resistance of a coating and the different degrees of hardness range from 0H the softest to 9H, the hardest. Coating with a high PFOTES-SiO_2_ ratio leads to reduced pencil hardness and adhesion of coatings due to the low surface energy of the PFOTES-SiO_2_ nanoparticles inducing a weak bonding strength between the nanoparticles and the matrix. Furthermore, the authors hypothesized that the dispersive/repellent nature of hydrophobic PFOTES-SiO_2_ nanoparticles may increase the number and volume of nanoscale pores in the composite, leading to reduced hardness and constant elasticity as PFOTES-SiO_2_ content increases.

**Figure 3:**
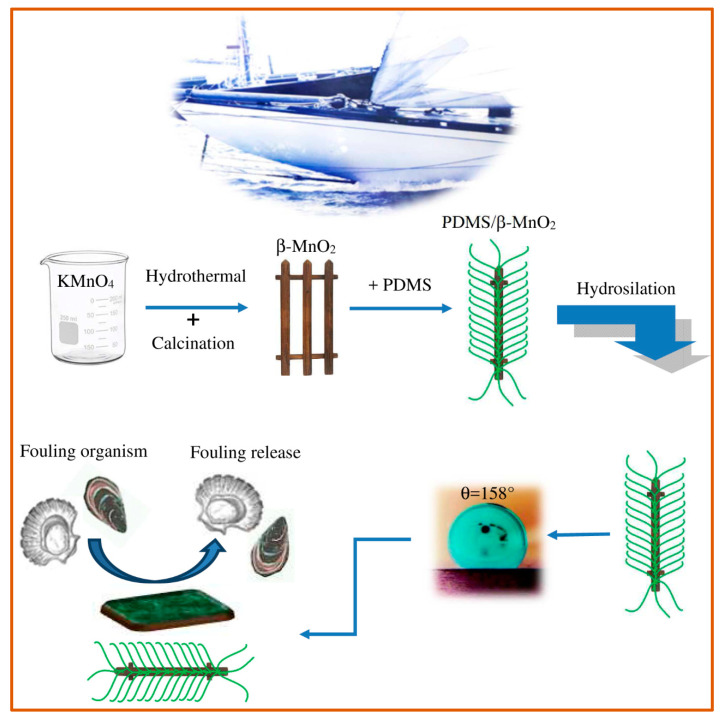
Synthetic route of polydimethylsiloxane (PDMS)/MnO_2_ coating with surface rough structure (re-organized from ref. [29]).

Nanocomposite systems, based on PDMS-nanorods of ZnO, β–MnO_2_ and GO-Al_2_O_3_ were investigated and characterized by WCA (water contact angle) static and dynamic (hysteresis) and surface’s mechanical properties (impact, mandrel bending, and cross-cut test) to check the coatings’ durability, adhesion strength to the substrate and elasticity [28,29,30]. Authors showed that each nanocomposite exhibited a nanorod optimum amount, within the range 0.5 wt% and 1.0 wt%. The different fillers in the silicone matrix can inhibit fouling growth due to the nanometric structures, its minimal free energy, and the superhydrophobic nature of the material. The excellent distribution of the nanorods in the silicone matrix leads, in fact, to a good interfacial bond between matrix and nanorods. The topology that is generated can trap the air between the drop of water and the surface of the nanocomposite. Therefore, the authors highlight that the realization of a rough surface, with low surface energy, is an excellent method to provide a superhydrophobic structure capable of inhibiting adhesion of fouling (Figure 3). These coatings also have high thermal, mechanical, and anti-corrosion stability over a wide pH range, while maintaining viscoelastic characteristics. At the same time, the authors point out that, at higher concentrations, the nanofillers (up to 5%) are no longer able to disperse well and form clusters; the nanorods grouping can cause the formation of voids, cracks, and/or holes in the matrix with consequent decrease in the adhesive power of the coating and in its performance against marine organisms.

A nanocomposite coating based on polydimethylsiloxane and ZnO (PDMS-ZnO) was also prepared by Arukalam et al. [31]. The further addition of per-Fluoro-Decyl-Trichloro-Silane (FDTS) to PDMS-ZnO allows to obtain an anticorrosive and antifouling coating. ZnO nanoparticles, due to antimicrobial properties and hydrophilic character, give antifouling activity and reducing hydrophobicity to the coating. FDTS was chosen to modify the surface energy of the coating and keep it within the Baier surface energy window for the application of the antifouling (i.e., within a range of 20–30 mN/m). In fact, it is known that these interactions are minimized by lowering the interfacial energy of the substrates through the presence of a low surface energy coating, such as FDTS. The optimal amount of FDTS of 0.4 g resulted in a decrease in the contact angle but improved antifouling and anti-corrosion characteristics. Surface energies, ranging from 26 to 30 mN/m, have proven effective for antifouling and anticorrosive applications on Q235 steel. The authors found that the anticorrosive efficacy of the PDMS-ZnO coating increases with the addition of FDTS. The adhesion strength (measured on a steel substrate by a pull-off adhesion tester) improves following the addition of FDTS: this is because the trichloro group (of the FDTS) increases the ability to form bonds with the hydroxyl groups on the surface of the steel. Thanks to the interaction of the trichloro group of FDTS with PDMS-ZnO, the material is more hydrophilic (in fact the static contact angle with water decreases as the FDTS increases). 

This agrees with the results of Neelakantan et al. [32]. Therefore, the authors demonstrated that corrosion protection is related to the surface chemistry and topography of the coating surface. Proper surface topography and low surface energy cause substrate surfaces to become anticorrosive and antifouling.

Wang et al. have studied a coating with ultra-heat resistant, adhesive, and anticorrosive properties consisting of polydimethylsiloxane (PDMS), furan, and meso-porous silica (SBA-15) [33]. The addition of SBA-15 in PDMS/furan provided excellent adhesive properties achieving a first level adhesive property in the cross-cut test compared to the third level of pure PDMS/furan coating. Furthermore, the presence of the filler SBA-15 in the matrix also allowed to gradually increase the angle of contact with the water, thus changing the surface from hydrophilic to super-hydrophobic. In fact, the contact angle in water reaches the value of 155.6°.

The well-known antimicrobial properties of silver have been exploited to synthesize an elastomeric nanocomposite made by polydimethylsiloxane (PDMS)/spherical Ag nanoparticles (NP, size < 10 nm). The coating has fouling release properties and it was applied using the solution casting technique on the first layer of epoxy primer and tie-coat layer after drying [34]. This modeling design proved effective for two inhibition effects: (1) chemical inertness and (2) physical repulsion of microfouling. The wetting behavior of the prepared (PDMS)/Ag-NP nanocomposites, shifted toward higher hydrophobicity up to 0.1% Ag and presented a contact angle of 148° ± 1° in water. The superhydrophobic surface nature could be attributed to the well-dispersed NPs that increased the surface area and smoothness and enhanced the chemical bonding between the NPs and the polymer matrix. The average surface roughness (Ra) measurements indicated that the microroughness value gradually decreased up to 0.1% Ag nanofillers (0.16 μm), resulting in ultrasmooth and non-stick surface. Higher concentration of Ag-NPs reduced the hydrophobicity because of enhanced agglomeration and particle clustering of Ag nanofillers. Cross-cut and impact test showed no changes in the bulk mechanical properties of nanocomposites up to 0.1% Ag with respect to pristine PDMS, indicating the high elasticity and flexibility of the PDMS nanocomposites.

The wettability and mechanical properties of siloxane matrices can also be adjusted by functionalization with organic parts, in addition to the inorganic fillers discussed above.

PDMS functionalized covalently with polyethylene glycol (PEG) or biocides were developed to obtain strong scale release or antifouling behavior, respectively. The former was synthesized as a copolymer of PDMS and PEG with hydrophilic and biocide-free scale release characteristics. Holberg et al. [35] investigated the antifouling and scale release performance of their coating versus commercial scale release for marine vessels using laboratory tests such as contact angle (CA), static and dynamic (advance-advance and recession-rec), pseudo barnacle PBT test. This last test is carried out by pulling aluminum pins, d = 20 mm at a speed of 0.2 MPa/s. The pins are glued onto the coated plates, using a two-component epoxy adhesive, cured for 10 min at 90 °C. The same authors found that the presence of 20% by weight of PEG makes the PDMS surface pure hydrophilic and reduces the growth and adhesion of scale (compared to steel) by a factor greater than 10. They also measured the contact angle, both static and dynamic, after dry and wet polymerization. They observed that the static contact angle after wet cure was greater than with dry cure, in both static and dynamic measurements. Furthermore, the contact angle value measured in advance was higher than the receding one (both in dry and wet curing) due to the presence of dynamic surface domains of a hydrophobic nature.

A silicone-based antifouling marine paint (PDMS) was obtained by covalently attaching two biocides, Irgarol (I) and Econea (E) through an isocyanate binder from Silva et al. [36]. In this case, the antifouling action occurs by contact because the biocide remains covalently bonded to the surface, in contrast to conventional release strategies. Crosscut tests have shown that all tested coatings, with or without biocides, can be classified between level 1 and 2, which corresponds to 5B and 4B (or ISO 0 and ISO1), for the standard ASTM3359-09e2 and ISO2409: 2013, respectively. This means that the percentage of the coating that is detached during the test varied from 5% to 15% and therefore the degree of adhesion is quite high. The contact angle of water on these silicone-based coatings is close to the value of 100°.

A copolymer based on an aromatic polyamide (HPA) and 3-chloropropyl trimethoxysilane (3CPTMS), with and without GO (graphene oxide), was developed by Arabpour et al. [37]. The presence of GO in HPA treated with silane had two effects: Act as a barrier against oxygen, water, etc., and improve the adhesion of HPA, with a consequent greater resistance to corrosion. The adhesion resistance of the coating was studied with the pull-off test (standard ASTMD4541). The aluminum dollies were glued with a double-sided adhesive on the surface of the covering and pulled. The hardness of the surface coatings was examined with the pendulum hardness (ISO standard 1522) until the coating was smooth by recording the frequency time. The progressive improvement of the adhesion strength for HPASi and HPASiGO (with 0.5% GO) compared to pure HPA is due to the strong interaction between polymer and substrate (through the chemical bond: Si–O–Fe) and between the nanoparticles of polymer and GO (Si–O–Si, Si–OC), which prevent the penetration of the corrosive electrolyte at the interface of the metal coating. The maximum strength and hardness of the HPA-Si-GO coating (with 0.5% GO) is due to the presence of rigid layered structures of the GO, and the presence of Si–O–Si networks.

In summary, in recent years, many attempts have been made to improve the mechanical properties and fouling resistance of silane-based coating. The proper combination of polysiloxanes matrix with organic or inorganic structures can allows to obtain coating with advantages in terms of smooth surfaces, ductile mechanical behavior, chemical stability, improved surface hardness, and hydrophobic character; further developments are necessary in order to overcome the main disadvantage of silane-based systems falling in low adhesion to steel substrate and low elastic modulus.

## 3. Epoxy Based Coatings

Epoxy coating acts as a barrier film, which protects and insulates the underlying metal marine structures against corrosive effect on metals due not only to oxygen and ion but also to microbiological fouling known as Induced Microbial Corrosion (“MIC”) [38,39,40,41]. The Authors cited in the following section studied the epoxy-based coatings for both anticorrosive and antifouling features. In fact, epoxy coatings can be used as a primer of metal substrates to protect against corrosive environments as well as epoxy topcoats containing biocide molecules can be used against marine fouling. Mechanical behavior was usually measured by means of a dolly-pull off adhesion test before and after immersion in NaCl solution or inside a saline fog chamber (salt test), to reproduce the marine environment. Table 2 resume all wet ability and mechanical adhesion test results of the epoxy-based coatings analyzed in this paragraph. 

Tian et al. 2015 [42] studied the failure behavior of epoxy coating, incorporated with inert pigments; marine environment was reproduced with a sodium chloride 3.5 wt% solution and under alternating hydrostatic pressure (AHP), in comparison with that under atmospheric pressure (AP) environment. 

They found that the AHP accelerates the failure of the coating with coating adhesion that decreases from 6.1 MPa to 4.1 MPa with the immersion time, compromising the protective properties of the coating. 

Correlations between adhesion and wet ability properties of epoxy coating containing different amount of Poly(m-aminophenol) (PmAP) or 2.0 wt% of bromo-substituted polyaniline (Br-PANI), named EBP were investigated by Quan et al. 2018 [43] and Cai et al. 2018 [44]. In particular, the coating studied by Quan et al. 2018 [43] exhibited high antifouling features and high corrosion protective performance shown by immersion tests in salt solution (12% NaCl). The contact angle of the epoxy coatings decreased from 79.8° to 70.1° with increasing of PmAP due to the presence of hydrophilic phenolic groups in PmAP microspheres. The adhesion resistance of the PmAP-based epoxy coatings (7.51 MPa) is considerably greater compared to the pure epoxy coating (3.36 MPa). Even after the 10-day salt treatment, the coating value remains always high (5.15 MPa). The increase in the adhesion strength may be due to chemical interaction involving the phenolic hydroxyl groups and the hydroxyl groups of the metal’s surface. 

High cross-linking density coatings based on epoxy matrix containing de-doped Br-PANI (EPB), investigated by Cai et al. 2018 [44], exhibited a better antibacterial and antifouling performance than pure references coatings that improved with the increase of the bromine content. The adhesion resistance of the EPB coating to steel has been tested before and during the immersion in 12.0 wt% NaCl solution at 95 °C. The dry adhesion strengths (0 day of immersion) of composite sample with code EBPIII resulted higher (5.90 MPa) than pristine epoxy and epoxy-PANI (EP) coating (3.54 MPa, 4.47 MPa, respectively). The authors asserted that the addition of Br has a positive effect on increasing the mechanical strength because PANI and Br-PANI can act as a bridge interconnecting epoxy resin and additives, leading to a reduced total free volume as well as an increase in the cross-linking density of the epoxy coating. Furthermore, the lone pair electrons of N and Br atoms in PANI and Br-PANI promotes the formation of coordination bonds with metal atoms in the metal/coating interfaces. After 25 days of immersion, the wet adhesion strengths of pure epoxy, EP, and EBP III decreased to 1.72 MPa, 1.80 MPa, and 3.75 MPa, respectively. Authors correlated this phenomenon to the coating’s wettability. The EP coating has the strongest wettability (60.2°) and absorbs much water that, consequently, compromises the adhesion strength between the coating and steel; on the contrary, EBP III coating has the lowest one (71.1°) showing the best protective properties that improve with the Br content.

P. Saravanan et al. [45] studied a high cross-linking network of nano-hybrid coatings based on tetraglycidyl 2,2-bis(4-(4-amine phenoxy)phenyl)propane (TGBAPPP), functionalized with 3-aminopropyltriethoxysilane (APTES) and ZnO as nano-reinforcement with antifouling and anticorrosion features. The nano-hybrid coatings were cured by aromatic and aliphatic curing agents. Cross-cut adhesion was carried out according to the standard method of ASTM 3359-83B (replaced by ASTM 3359-09) [46] and the best results were obtained for ZnO-APTES loaded TGBAPPP with curing agent (which is Aradur 140). The presence of ZnO and APTES acts as nano-structured cross-linking sites to form coatings with high cross-link density resulting in tough and relatively hard protective films to metals. 

Mohammadkhani et al. [47] studied an epoxy resin mixed with graphene oxide (GO) nano-platforms, polypyrrole (PPy) and zinc metal ions, for anti-corrosion functions. PPy nanoparticles and zinc cations make the GO nanosheets more compatible with epoxy coating. Pull-off test evaluated the adhesion of neat epoxy coating and the nanocomposites, under dry and wet condition after salt spray test for 400 h. Incorporation of nanoparticles into the epoxy coating enhanced the resin’s adhesion forces both under dry and wet conditions. The sample reinforced by GO-PPy-Zn exhibited the highest adhesion strength thanks to the surface modification of GO nanosheets with PPy and Zn, which improves the interfacial adhesion between the epoxy resin and the steel substrate.

The cross-linked final structure of epoxy resins ensures lower water permeability coefficient, good adhesion, and mechanical performance but too high cross-linking density can be detrimental due to too high stiffness and low impact resistance. To overcome this drawback, the epoxy resin can be suitably modified with siloxanes [48]. Epoxy and silicone are complementary materials in terms of mechanical and chemical-physical properties (such as adhesion, permeability, and rigidity). The addition of silicone to the epoxy resin effectively decreases the adhesion and the mechanical properties of the coating, but it enhances the ductility and surface characteristics typical of the silicone.

Among the various types of antifouling and/or anticorrosive coatings, silicone-based ones have low surface energy and are not polar. The adhesion of marine organisms on their surface is therefore strongly hampered due to their peculiar hydrophobic character. The low adhesion to the metal substrate and poor mechanical properties limit the application of silicone coating on metallic surfaces. Thus, epoxy-silicone material can be well designed to have an adaptable cross-link density to balance the rigidity of the epoxy resin and the elasticity of the silicone material. The balance of epoxy resin and siloxane is the key factor for a suitable coating in terms of adhesion, hydrophobic characteristics, and mechanical resistance and toughness [2,49]. Finally, the addition of proper fillers (such as graphene, ZnO, etc.) to epoxy-silicone coating is considered to further improve some peculiar characteristics such as the control of the adhesion towards the metallic or composite substrate. 

Sun et al. [2] synthesized an epoxy modified poly-siloxane based resin (code: EAPDMS) in two steps: Polydimethylsiloxane (PDMS) has been synthesized with aminopropyl-terminated pendant groups (APDMS), which then reacted with bisphenol A type epoxy resin (DGEBA). Mechanical features highlighted that the epoxy resin presence improves the material’s stiffness up to 303 MPa in EAPDMS sample with 70.5 mmol of APDMS and 35.2 mmol of DGEBA. An increase of DGEBA content up to 46.2 mmol maintaining the APDMS/DGEBA ratio fixed to 2:1 lead to a decrease in mechanical features due to the lower cross-linking density of the modified system and to the lower strength of APDMS molecular chain. The coating’s pull-off strength was evaluated on a glass fiber reinforced epoxy resin panel, according to the ASTM D4541-09 standard. The low adhesion strength of silicone (0.4 MPa) increased more than two magnitude order with the epoxy content (up to about 8 MPa in the sample with the highest DGEBA content) because of the increase in hydroxyl functional groups generated after the opening of the epoxide ring. Therefore, the addition of DGEBA increases the crosslinking density of the material, visibly improving both the mechanical properties and the chemical compatibility, and therefore the adhesion of the coating towards the composite laminate. R. Zhao et al. [49] designed a modified three-component epoxy silicone resin with the aim to improve the mechanical performance and the adhesion of the silicone antifouling coatings. The results show that the presence of epoxy resin improves the adhesion of the coating up to level 1, while the free energy of the coating surface is between 15 mJ/m^2^ and 21 mJ/m^2^. If the amount of epoxy is lower than 22.1 wt%, the coating has ductile characteristics, but if it is higher than this threshold, it becomes fragile and an increase of the hardness (and stiffness) and fracture strength and a decrease of bacteria removal properties were observed.

**Figure 4:**
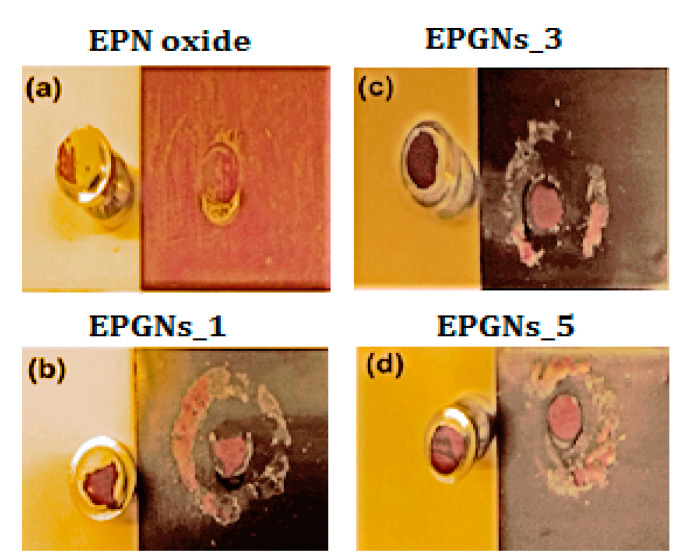
Digital images of adhesion pull off test results in the Verma et al. nanocomposite coating EPN oxide(**a**), EPGNs-1 (**b**), EPGNs-3 (**c**), EPGNs- 5(**d**) (Adapted with permission from [50]).

Other authors added specific fillers to epoxy modified poly-siloxane based resins to improve their features. Verma et al. [50] incorporated different amount of graphene oxide nanosheets (GNs) into epoxy-hydroxy-terminated-polydimethylsiloxane (EP-hPD) matrix to increase the modulus of elasticity and tensile strength and the water contact angle (from 90.1° to 115.2° at 1.0 wt.% amount of GNs). This enhancement in adhesion and wettability properties of nanocomposite is due to the synergistic effect of GNs within the polymeric matrix. Figure 4 shows the digital images of the pull-off test of epoxy-silicone-GNs nanocomposites in which the adhesion progressively improves, leaving the black coating attached in the metallic dolly.

Stojanovic et al. [51] evaluated the protective properties of two different anticorrosion–antifouling coatings: The first was self-polishing system (based on copper, codified as AF1), and the second was a new fouling-release one, based on miscellaneous components (codified as AF2). They tested both coatings in different conditions: Humid and warm atmosphere, and in a simulated marine environment (in a salt spray chamber, with and without agitation). The best pull-off adhesion resistance was obtained after the immersion test with agitation.

In summary, as discussed above and resumed in Table 2, epoxy-based coatings exhibit a lower hydrophobic character and higher mechanical performance, higher adhesion, and higher stiffness with a lower tensile strength, compared to silane-based ones. Thus, their great advantage is the particularly good adhesion especially with metallic substrates, while their disadvantage is the poor ductility.

## 4. Polyurethane Coatings

Fouling-release technology needs to be applied to fast-moving (>15 knot) vessels with high mechanical stress on the coating film. Although PDMS-based coatings exhibit the desired properties to prevent fouling attachment, they suffer from some disadvantages in terms of mechanical properties and, for these reasons, there has been extensive research on newer fouling-release technologies. 

Coatings based on polyurethane matrix mixed with antifouling filler or obtained by copolymerization have been investigated. Table 3 resume all wet ability and mechanical adhesion test results of the polyurethane coatings analyzed in this paragraph.

Degradable polyurethane-based coatings were investigated by two-step condensation polymerization of poly(propylene carbonate) polyurethane (PPCU) [52] or by mixing triblock polyols (polyether (polypropylene glycol or polyethylene glycol) and ε-caprolactone) and crosslinker agent (toluene diisocyanate trimer) [53]. The hydrolytic degradation of the polyurethane coating allows a self-renewal antifouling mechanism able to release foulants entrapped in the polymeric matrix. The polyurethane films show hydrolysis rate (0.012–0.051 g/(m^2^d)) that increases as the molecular weight of polymeric components decreases; polyurethane coatings usually also possess good resistance to fracture with impact resistance of 50 kg·cm/cm.

The effect of different amount of PDMS inside a polyurethanic matrix was investigated by Zhang et al. [54]. The increase of PDMS content in the polyurethane matrix increased the phase separation and the surface roughness. In addition, the hybrid systems showed an increase of tensile strength and elastic modulus with respect to pure polyurethane.

The proper control of the chemical structure of polyurethane in terms of ratio of soft and hard segment was also used to tune the antifouling property of the coating. In particular, synthesis of polyurethane antifouling coating using 2,4-toluene diisocyanate and 1,4-butanediol as the hard segment and polyether polyols (polypropylene glycols (PPG) as the soft segment allows to obtain antifouling behavior with water contact angle of 94° and elastic modulus that increases rapidly with increasing hard segment contents (up to 260 MPa); this behavior was related to strongest interaction force among the segments as a result of the increase in the hydrogen bonds and crosslink density [55].

The effect of fillers insertion in the polyurethane matrix was also investigated; nanocomposites based on ZnO and gamma-irradiated multiwall carbon nanotubes (MWCNT) mixed with polythiourethane or polyurethane, respectively, showed an increase of the mechanical properties in terms of tensile, hardness, and pull-off test results [56,57]. The presence of MWCNT led to the formation of a high degree of crosslinking and hydrogen bond between irradiated MWCNT and polyurethane chains obtaining an increase in the pencil hardness values up to highest value (7H) and improvement in adhesion strength up to highest value (5B). 

Silva et al. [36] covalently attached two biocides, Irgarol (I) and Econea (E) to polyurethane (PU) paints through an isocyanate linker. The obtained average pull-off forces of polyurethane coated surfaces were about 3.1 MPa, and no significant change was observed after the immobilization of biocides in the polyurethane matrix; also, no detrimental effect due to the presence of the biocides was observed in terms of adhesion strength (level 1 and 2 in cross-cut test) and contact angle of water (close to 75° and 85°).

In summary, as discussed above and resumed in Table 3, polyurethane-based coatings exhibit a low hydrophobic character and intermediate mechanical and adhesion features, compared to these of the silane-based and of the epoxy-based ones. This class of coating exhibits particularly advantages in terms of self-renewal antifouling behavior due to hydrolytic degradation of the polyurethane matrix.

## 5. Other Systems

Other systems, whose chemical nature is different from the epoxy-, silane-, and polyurethane-based coatings, have been collected in this chapter. Table 4 presents all wet ability and mechanical adhesion test results of the other coating systems analyzed in this paragraph.

Acrylic-based coating attracted also great attention due to high performance in terms of mechanical properties. Hydrophobic or superhydrophobic coatings have been developed by using polyacrylate resins modified with fluorine or polysiloxane or loaded with reduced graphene oxide and cuprous oxide. The increase of the amount of fluorine containing monomers with long flexible groups leads to an increase of the pencil surface hardness values (4H or 5H) and water contact angle (around 109°) [58]. Condensation reaction of polyacrylate with polysiloxane leads to a hydrophobic, 6H pencil hardness and 5B cross-cut adhesion copolymer where the presence of acrylic chain ensures strong adhesion to substrate while the side-chain Si–O bonds from polysiloxane enable the copolymer to hydrolytically degrade so providing a self-polishing character with water contact angle equal to 98°. A series of fluorinated/silanized polyacrylates amphiphilic polymers were successfully synthesized by Zhu et al. [59] and the effect of the loading of fluorinated groups content was investigated. With the increase of fluorinated groups content, water contact angle increased from 85° in the fluorine-free polymer to 116.3° in the fluorinated ones. All polymers exhibit excellent adhesion to steel and glass and hardness of 3H or HB. The adhesion strength is slightly reduced (from 5B to 4B) for high fluorine content (21.4 wt% and 15.4 wt% for steel and glass, respectively). Sun et al. [60] studied the surface energy, mechanical properties, and adhesion of crosslinked network coatings based on α,ω-triethoxysilane (PDMS) terminated perfluoropolyether (PFPE) oligomer and acrylic polyols (codified as PFPE/PDMS/AOH). Experimental results shown that these materials possessed good adhesion (thanks to the siloxane functional group), low surface energy (generally within the range 19.3–21.7 mJ/m^2^), and low elastic modulus (generally within the range 19–47 MPa).

Addition of reduced graphene oxide to acrylic matrix increased the water contact angle from 45° to 113° and ensured an adhesion between marine antifouling coating and intermediate coating equal to 5B while the average adhesive force measured by pull-off tests was 3.69 MPa [61]. Copolymer systems based on PVA and MMA functionalized with three different monomers, amine (acrylamide), carboxylic (acrylic acid), and hydroxyl (ethylene glycol), were developed by Ghani et al. [62]. Copolymer functionalized with carboxylic groups showed the highest adhesion strength of 4.0 MPa on carbon steel plate while functionalization with hydroxyl groups lead to adhesion values lower than the unfunctionalized copolymer (respectively, 1.3 MPa than 2.07 MPa).

Multilayer polymers with antifouling properties were synthesized by using 3-aminopropyltriethoxysilane and dopamine (HPAPD). A hydrophilic coating was investigated by Meng et al. [63] where a substrate of stainless steel (SS) was first coated with a hybrid polymer film, which was formed by simultaneous hydrolytic polycondensation of 3-aminopropyltriethoxysilane, polymerization of dopamine (HPAPD) and grafting of PMMA-b-PHEMA (poly(methyl methacrylate) and poly(2-hydroxyethyl methacrylate). The presence of HPAPD make the sample hydrophilic reaching water contact angles from 92° for uncoated stainless steel to 52° for stainless steel coated with the block copolymer (SS-HPAPD-PMMA-b-PHEMA). On the other hand, hydrophobic multilayer coating was synthesized grafting p-phenylenediamine (PPD) to the SS–HPAPD substrate; the amino groups of p-phenylenediamine were further used as anchors for the growth of polyaniline (PANA) nanofiber arrays by polymerization of aniline in situ. The nanofibers were further silicificated using 3-aminopropyltriethoxysilane (APTS), 3-mercaptopropyltriethoxysilane (MPTS), vinyltrimethoxysilane (VTMS), and octyltrimethoxysilane (OTMS) conferring various functional groups [64]. The polyaniline-based coating showed an increase of hydrophobic character with water contact angle from 92° on untreated SS to 102° on SS-HPAPD-PPD-PANA system. The silicification led to a further increase of hydrophobic behavior with water contact angle of 132° for SS-HPAPD-PPD-PANA-OTMS system.

The development of antifouling coatings with hydrophobic character was also focused in recent years on “non-conventional” polymers. Barroso et al. [65] investigated coatings based on pure polysilazane or loaded with poly(tetrafluoroethylene) (PTFE) particles. Such systems possess a higher amount of nonpolar organic groups resulting in a low surface free energy that was further lowered by the addition of PTFE fillers. Unfilled coatings showed water contact angle equal to 86.8° that shifted to 114° with PTFE loaded polysilazane systems. Pull-off test showed adhesion of 12.7 MPa for the unfilled system, which decreased to 4 MPa for the PTFE filled polysilazane systems. Ren et al. [66] prepared self-regenerating functional coatings, by layer-by-layer self-assembly method, composed of chitosan and dialdehyde starch. The authors evaluated the adhesion of the coating with the shear test on different substrates (plastic, acrylic, glass, metal, ceramic), and by paint adhesion testing (cross-cut test). In general, the materials showed good adhesion with all substrates except the metal one. Fu et al. [67] prepared nanocomposite coating with a dual hierarchical structure, composed by fluorinated copolymer functionalized with quaternary ammonium salts and fluorinated copolymer functionalized with poly(urea-formaldehyde) nanoparticles. Due to a synergistic effect of fluorinated segments and quaternary ammonium ions, the coatings showed a super hydrophobic character and marked antibacterial performance.

In conclusion, hybrid systems aim to have a high adhesion towards metal and composite substrates with a polymeric matrix, reaching a medium-high value of hydrophobicity, depending on the composition.

## 6. Conclusions and Future Perspectives

Antifouling coatings are essential to prevent scale growth on submerged structures. There is a long history behind their development involving enormous technological and research advancement in order to replace the heavy metals and toxic biocides based antifouling paints. A winning strategy can be represented by the development of multifunctional coatings capable of combining different design properties in a single coating. This often requires a complex balance between the hydrophilic/hydrophobic components, good adhesion to the substrate, ease of application.

This review summarized the recent advances in terms of mechanical properties of protective coatings based primarily on silane and epoxy resins. Polyurethanes and hybrid materials or “other systems” than the main ones were also considered.

During this review we have shown that the scientific community has paid great attention mainly in order:

- To modulate the wet ability of the coating which is important in terms of repulsion against marine fouling;
- To study the mechanical behavior because the ideal coating must have the best adhesion to the support, both metallic and polymeric composites.

Nowadays, epoxy- and silicone-based coatings represent the main class of coating investigated; epoxy coatings suffer of a low hydrophobic character with high adhesion towards metallic and polymeric composites substrates; on the other hand, silicone-based paints exhibits high hydrophobicity and low adhesion. Similarly, polyurethane-based coatings, and more generally the hybrid systems, show high adhesion and low hydrophobicity and vice versa, depending on their composition. The best paints should represent a compromise between these two properties by adding specific fillers and/or by combining different polymeric materials, suitably chosen.

From what emerged in this review, some possible directions for future research in this field are as follows:

- A self-layers coatings could represent a valid alternative because they are multilayer or gradient coating structures [68]. In this way, the different layers of coatings (hydrophobic, amphiphilic, super hydrophobic, or super hydrophilic) should be designed in the customization of their characteristics to be applied in the specific case.
- A self-renewal coatings with optimized properties in terms of degradation and mechanical behavior.

The development of this kind of coatings requires an interdisciplinary approach in which biology, chemistry, and materials engineering may be able to design materials with adequate properties of both adhesion to substrates (mechanical properties) but also resistance to marine fouling. These environmentally friendly solutions must be easily and economically transformed into industrial scale-ups for large-scale commercialization.

## Figures and Tables

**Table 1:** Contact angle and mechanical characterization of silane-based coatings.

Ref.	Coating Type	Contact Angle (Water)	Crosscut Adhesion (ASTM3359-09e2)	Pull/Off Adhesion (MPa)	Impact Test (J)	Cross Hatch	Bending Test (mm)
[25] ^1^	fluoroalkyl silane (FAS)/CeO_2_	161 ± 2°	4B _(level 2)_		-	-	-
[26]	PDMS/TiO_2_	10° ^2^	-		10	pass	<5
[27] ^3^	Perfluorooctyltriethoxysilane (PFOTES)-SiO_2_	166° ^4^	5B	6.85 ± 0.16	-	-	-
[28]	PDMS/ZnO NRs (0.5 wt%)	158°	-		15	pass	<5
[29]	PDMS/b-MnO_2_ NRs (0.5 wt%)	158°	5B		15	pass	<5
[30] ^5^	PDMS/GO-Al_2_O_3_ NRs (1.0 wt%)	151°	-		15	pass	<5
[31,32]	FDTS-PDMS-ZnO	120.64°	-	2.41 ± 0.03	-	-	-
[33]	polydimethylsiloxane (PDMS), furan and meso-porous SiO_2_ (SBA-15)	155.6°	5B _(level 1)_		-	-	-
[34] ^6^	PDMS/Ag	148° ± 1°	-		8	pass	<5
[35]	PDMS-PEG 20%	117°	-		-	-	-
[36]	PDMS	100°	4/5B _(level 2/1)_		-	-	-
[37]	HPA-Si-GO	-	-	1.25 ± 0.07			

^1^ sliding angle: first cycle 4°, after 30 cycles of abrasion < 6°. ^2^ After UV irradiation. ^3^ Surface roughness: 2–3 μm, Pencil hardness: 8H (being the grades from 0 to 9 H:hardest), Hardness: 0.24 ± 0.01 GPa. Modulus: 1.57 ± 0.11 MPa. ^4^ 105° in dodecane. ^5^ Modulus: 4.12 ± 0.1 MPa. ^6^ Surface roughness: 0.16 micron.

**Table 2:** Contact angle and mechanical characterization of epoxy-based coatings.

Ref.	Coating Type	Contact Angle (°)	Adhesion Test Dolly-Pull Off (MPa)		Mechanical	
		Water	Before Salt Test	After Salt Test	Modulus (MPa)	Strength (MPa)
[2] ^1^	Silicone	-	5.2 ± 0.5	-	303.4 ± 0.4	28.5 ± 0.9
[42]	inert pigments	-	6.1	4.1	-	-
[43]	Cardanol Phenalkamine	70.1	7.51	5.90	-	-
[44]	Br/Pani	71.1	5.90	3.75	-	-
[45]	ZnO-APTES	-	5B ^(crosscut adhesion)^	-	-	-
[46]	Go/PPy/Zn	-	5.42	1.5	-	-
						
[47] ^2^	Silicone	113.17± 1.33	-	-	2.02 ± 0.23	0.24 ± 0.02
[48] ^3^	Silicone/GNs	115.2	5.94 ± 1.17	-	1570	31.54
[49]	Silicone-Acrylate/Cu ^(self polishing)^	-	1.435 ± 0.245	-	-	-

^1^ Fracture elongation (%) = 92 ± 7 and 47 ± 4, Toughness = 7.6 ± 0.1 (J/m^3^) and 0.8 ± 0.4 (J/m^3^). ^2^ Shore D Hardness = 80.20 ± 3.06; Adhesion level = 5B; Fracture elongation (%) = 17.88 ± 0.24. Impact resistance=50 Kg × cm. ^3^ Abrasion resistance test: weight loss (mg/1000 cycle) = 3.4 ± 1.14.

**Table 3:** Contact angle and mechanical characterization of polyurethane-based coatings.

Ref.	Coating Type	Contact Angle (Water)	Dolly-Pull Off Adhesion Test (MPa)	Hardness (GPa)	Modulus (GPa)	Tensile Strength (MPa)	Elongation at Fracture (%)
[36] ^1^	polyurethane	75-85	3.1	-	-	-	-
[52,53] ^2^	polyurethane	-	7.2	-	0.07	11.63	65
[54] ^3^	polyurethane/ polydimethylsiloxane	106	-	-	-	-	-
[55]	polyurethane modified with hard//soft segment (PPG-TDI-BDO)	94	-	-	0.26	-	14
[56]	polythiourethane (PTU)/ZnO	72.8 ± 3.3	0.64 ± 0.04	81.0 ± 1.6	0.952 ± 0.083	58.7 ± 1.4	12.1 ± 0.5
[57]	polyurethane/ multiwalled carbon nanotubes	-	5B	7H	-	-	-

^1^ Cross cut adhesion: level 1–2. ^2^ Impact test: 4.9 J. ^3^ Modulus: 1.3 MPa, Tensile strength: 2.09 MPa, Elongation at fracture: 1315%, Surface roughness: 0.1 micron (Rz), 0.02 micron (Ra), contact angle in diiodomethane: 63°.

**Table 4:** Contact angle and mechanical characterization of other coating systems.

Ref.	Coating Type	Contact Angle (Water)	Cross Cut Adhesion (ASTM3359-09e2)	Dolly-Pull Off Adhesion Test (MPa)
[47] ^1^	polyacrylate-g-polysiloxane	98°	5B	-
[58] ^2^	urethane methacrylates	109°	5B 5B	-
[59] ^3^	fluorinated/silanized polyacrylates amphiphilic polymers	116°	5B	-
[60] ^4^	PFPE/PDMS/acrylic	107 ± 0.6	5B (on glass)	-
[61]	acrylic resin loaded with reduced graphene oxide and cuprous oxide	113	5B ^(Class 1)^	3.69
[62]	Copolymer systems based on PVA and MMA	-	-	4
[63]	HPAPD-PMMAb-PHEMA (dopamine, poly(methyl methacrylate) and poly(2-hydroxyethyl methacrylate)	52	-	-
[64]	dopamine (HPAPD), p-phenylenediamine (PPD), polyaniline (PANA), 3-aminopropyltriethoxysilane (APTS), 3-mercaptopropyltriethoxysilane (MPTS), vinyltrimethoxysilane (VTMS) and octyltrimethoxysilane (OTMS)	132	-	-
[65] ^5^	polysilazane loaded with poly(tetrafluoroethylene) (PTFE) particles	86°	5B	12.7
[66] ^6^	Chitosan + starch	-	2B	-
[67] ^7^	Fluorinated copolymers and poly(urea-formaldehyde)	162.3 ± 3.2	-	-

^1^ Surface roughness: 0.068 micron, Pencil hardness test: 6H. ^2^ Pencil hardness test: 4H, Contact angle in hexadecane: 59°. ^3^ Hardness: HB. ^4^ Modulus: 22.16 MPa. ^5^ Contact angle in diiodomethane: 49.8°. ^6^ Lap shear test: on Metal 38.3 N, on plastic 200 N, on glass plastic, acrylic, ceramic 4B, on metal 2B. ^7^ Contact angle after 16 abrasion cycles: 150 ± 3.2, Cross cut adhesion test: good on glass after 20th cross cut tape test.

## Data Availability

Data are contained within this article.
